# The role of cellular senescence in cardiovascular disease

**DOI:** 10.1038/s41420-025-02720-5

**Published:** 2025-10-06

**Authors:** Chengying Xu, Zhimei Qiu, Qing Guo, Youyang Huang, Yongchao Zhao, Ranzun Zhao

**Affiliations:** Department of Cardiology, Affiliated Hospital of Zunyi Medical University, Zunyi, China

**Keywords:** Senescence, Atherosclerosis

## Abstract

The incidence of cardiovascular diseases rises significantly with age, making it one of the leading causes of death and disability worldwide, and cellular senescence plays a crucial role in this process. Cellular senescence constitutes a salient feature of organismal aging and stands as an independent risk factor for a range of cardiovascular diseases, encompassing hypertension, atherosclerosis, myocardial infarction, heart failure, and arrhythmia. This comprehensive review endeavors to comprehensively delineate the intricate regulatory mechanisms underlying cellular senescence and its attendant biological implications, while elucidating the profound implications of this process on the initiation and progression of cardiovascular diseases. Finally, we will delve into a spectrum of targeted interventions aimed at cellular senescence, specifically focusing on eliminating the accumulation of senescent cells during disease progression or inhibiting the inherent cellular senescence processes. Our ultimate goal is to mitigate or postpone the onset of diseases that are intricately linked to cellular senescence. A profound comprehension and rigorous investigation into the regulatory mechanisms of cellular senescence and their intricate interrelationships hold significant potential to furnish invaluable scientific evidence for the prevention and therapeutic strategies against cardiovascular diseases.

## Facts

- Cellular senescence promotes the occurrence and progression of cardiovascular diseases such as hypertension, atherosclerosis, and myocardial infarction through multiple molecular mechanisms (e.g., SASP secretion, oxidative stress, mitochondrial dysfunction), serving as a critical independent risk factor for these conditions.
- Senolytics (e.g., dasatinib, quercetin, ABT-263) can remove senescent cells and improve myocardial remodeling and renal function; Senomorphics (e.g., rapamycin, metformin) reduce inflammation by inhibiting SASP secretion or related signaling pathways (e.g., mTOR, SIRT1).
- Single-cell sequencing technology revealed significant heterogeneity of senescent cells across different organs, with their phenotypic and functional differences potentially influencing the selection of intervention strategies.
- Metabolic disorders (e.g., decreased NAD levels, hyperglycemia) and epigenetic modifications (e.g., DNA methylation, miRNA expression) can drive cellular senescence and form a metabolic-aging-disease vicious cycle.

## Open questions

- Do different senescent cell subsets (e.g., pro-inflammatory, reparative) respond differently to the same intervention? How can subpopulation-specific targeting be achieved to improve treatment outcomes?
- Removal of senescent cells may affect tissue repair function. How to balance the efficacy with the potential risks? What clinical trial designs are needed to validate its safety?
- Can the integration of single-cell data with epigenetics, metabolomics, and other multi-omics reveal the core regulatory networks that drive aging? How to overcome technical challenges to drive clinical translation?

## Introduction

Cardiovascular disease poses a profound global concern for public health, with age standing as a crucial risk factor that contributes significantly to the progressive deterioration of cardiac structure and functionality [1]. As the trend of population aging intensifies, age-related diseases, including those affecting the cardiovascular system, are increasingly becoming prevalent. Among the elderly population, age-related diseases represent a substantial contributor to elevated hospitalization and mortality rates, thereby significantly augmenting economic burdens [2].

Aging is a biological process characterized by the gradual decline in cellular and organismal functions over time [3]. resulting in an increase in age-related diseases. It is a primary risk factor for a multitude of conditions, including cardiovascular diseases (e.g., hypertension, heart failure, myocardial infarction), neurodegenerative diseases (e.g., Alzheimer’s disease), cancer, and diabetes [4]. In mammals, the aging process is linked to the build-up of senescent cells. During aging, cells undergo mitochondrial dysfunction, DNA damage, and increased activation of the p53/p21 and p16 signaling pathways in response to cellular stress, ultimately contributing to the development and advancement of cardiovascular diseases [5]. Cellular aging plays a crucial role in the pathogenesis of heart disease [6]. In recent years, cellular senescence has garnered considerable interest as a potential target for alleviating age-related diseases and extending lifespan. Cellular senescence is a hallmark of aging, characterized by a stable cell cycle block accompanied by typical morphological changes in cells and a distinguishable secretory phenotype [7]. The aim of this review is to provide a comprehensive summary of the role of cellular senescence in cardiovascular disease and related mechanisms. To begin, an overview of the fundamental concepts, characteristics, and biological effects of cellular senescence will be provided. Additionally, we will delve into the regulatory mechanisms of cellular senescence, encompassing the key molecules and signaling pathways involved. Subsequently, our focus will shift to exploring the interconnections between cellular senescence and conditions such as hypertension, atherosclerosis, myocardial infarction, heart failure, arrhythmias, and cardiomyopathy. This exploration aims to illuminate the role of cellular senescence in the development and progression of these diseases. Finally, we will also explore the impact of targeted cellular senescence-related therapies on the aforementioned cardiovascular diseases. Achieving healthy aging has become a formidable challenge in modern society; therefore, the exploration of the role of aging is crucial for the prevention and amelioration of age-related diseases. A profound comprehension of the roles and mechanisms of cellular senescence in cardiovascular diseases aims to provide new ideas and strategies for the prevention and treatment of cardiovascular diseases.

## Basic concepts and mechanisms of cellular senescence

### Definition, classification, and characterization of cellular senescence

Cellular senescence is a response triggered by acute or chronic injury [8]. It is characterized by a combination of stable cell cycle arrest and distinct phenotypic changes [9–11]. This phenomenon was initially observed in 1961 by American biologist Leonard Hayflick, who cultured normal human fibroblasts in vitro. Even under optimal growth conditions, these cells eventually reach a limit in the number of divisions they can undergo, leading to cell cycle disruption and entry into an ‘irreversible’ state of stagnation. It was from this observation that Hayflick first introduced the concept of cellular senescence [12]. Cellular senescence in the cardiovascular system is triggered by a combination of internal and external factors such as telomere dysfunction, persistent DNA damage, activation of oncogenes, oxidative stress, and mitochondrial dysfunction [7]. On the other hand, cellular aging is caused by factors including activation of internal signaling pathways, free radical damage, changes in gene expression, and changes in the extracellular matrix environment. These factors can affect the life cycle and function of cells, causing affected cells to gradually lose their normal structure and function and eventually die. In summary, cellular senescence includes primary cellular senescence caused by factors such as oncogenic signaling, genotoxic damage, telomere attrition, mitochondrial dysfunction, viral or bacterial infection, oxidative stress, nutritional imbalance, and mechanical stress [8], and secondary or paracrine cellular senescence triggered by extracellular mediators related to inflammation and fibrosis, such as CCL2, IL-1β, IL-6, IL-8, and TGF-β [13].

**Fig. 1:**
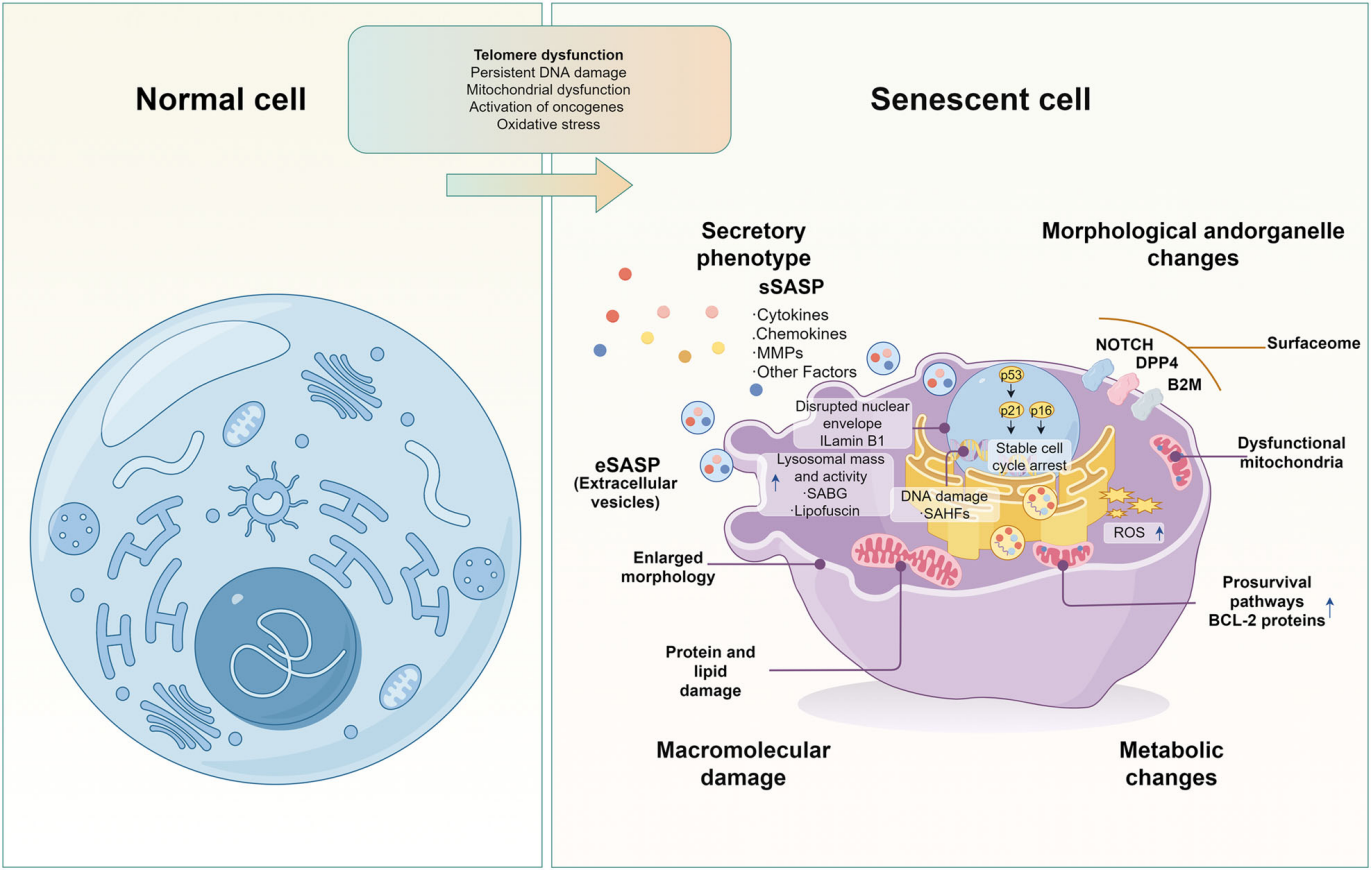
Characteristics of normal cells vs. characteristics of senescent cells. The characteristics of normal cells include: normal cell morphology, clear boundaries, intact structures of the cell nucleus and cell membrane, and evenly distributed and moderate numbers of intracellular organelles such as mitochondria, Golgi apparatus, and endoplasmic reticulum. Senescent cell features include: The prolonged arrest of the cell cycle is achieved through the upregulation of the p21 and p16 cell cycle inhibitors. The oxidative damage is identified by the elevation in reactive oxygen species (ROS) levels. The upregulation of the BCL-2 family of antiapoptotic proteins induces resistance to apoptosis. Senescence-associated heterochromatin foci (SAHF) and a senescence-associated secretory phenotype (SASP). Metabolic changes, which encompass the accumulation of senescence-associated β-galactosidase (SA-β-gal), are evident. Morphological changes. A signaling cascade, known as the DNA damage response (DDR), can be defined as a series of events triggered by DNA damage.

The distinctive feature of cellular senescence is its irreversible cell cycle arrest in the G1/S or G2 phase, resulting in a permanent exit from potential proliferation, resulting in a gradual decrease in cell growth, differentiation, and biological activity [14, 15]. The distinguishing feature of cellular senescence is its irreversible cell cycle arrest in the G1/S or G2 phase, leading to a permanent withdrawal from potential proliferation, which results in a progressive decrease in cell growth, differentiation, and biological activity [16]. Although there are many triggers that cause cellular senescence and the molecular mechanisms that mediate senescence in different types of cells vary widely, cells that undergo senescence still exhibit a series of similar and specific features that include: Prolonged cell cycle arrest, oxidative damage, up-regulation of the BCL-2 family of anti-apoptotic proteins, alterations in cellular morphology, nuclear senescence-associated heterochromatin foci (SAHF), senescence-associated secretory phenotype (SASP), metabolic changes, DNA damage response [15, 17, 18] (Fig. 1). Numerous studies have demonstrated that the phenotype of senescent cells is highly heterogeneous and dynamic, a phenomenon that may result from different senescence processes. The senescent phenotype usually exhibits the activation of chronic DNA damage response accompanied by the involvement of various cell cycle protein-dependent kinase inhibitors, enhanced secretion of pro-inflammatory and tissue remodeling factors, induction of anti-apoptotic genes, metabolic alterations, and endoplasmic reticulum (ER) stress; the morphological changes of senescent cells are manifested by extensive vacuolization, increase in cell size, irregular shape, increase in the lysosomal content and disruption of nuclear membrane integrity [19] (Fig. 1). Taken together, these features or combinations of related phenotypes and markers can be used together as criteria for determining cellular senescence.

### Biological consequences of cellular senescence

#### Physiological functions and pathological roles of cellular senescence

Not all senescent cells exhibit identical phenotypes; there is substantial heterogeneity among them. Consequently, the biological consequences of cellular senescence are dualistic, with the process exerting multifaceted functions under different physiological and pathological conditions. Their beneficial effects are manifested as follows: first, senescent cells form temporary structures by secreting FGF4 and FGF8 to guide embryonic tissue regeneration and embryonic development, and shape placental structure and function together with matrix metalloproteinases 2 and 9. Second, senescent cells limit excessive cell proliferation to avoid further tissue damage and also promote wound healing by secreting PDGF-AA. In addition, senescent cells autonomously impede cell cycle progression by up-regulating the expression of genes such as p53, p16, and p21, thereby limiting tumor growth. At the same time, senescent cells also secrete cytokines such as IL-6 and IL-8 to promote senescence of surrounding cells, thereby limiting tumor spread in a non-autonomous manner [16, 20–24]. Thus, cellular senescence is an effective tumor suppressor mechanism. Finally, a large body of evidence suggests that cellular senescence is associated with the process of tissue repair, in which senescent cells promote local fibrosis and recruitment of immune cells, followed by removal of damaged and senescent cells [25]. On the other hand, the deleterious effect of senescent cells is that they promote a sterile chronic inflammatory response by secreting a variety of senescence-associated SASP components, such as the secretion of IL-6, IL-1 receptor antagonist (IL-1RA), GROα and IFNγ, and MMPs, thereby destroying tissue structure and promoting tumor growth [26, 27]. SASP is a paracrine reservoir of pro-inflammatory cytokines, chemokines, growth factors, and proteases [17, 28]. Paracrine activity of senescent cells plays an important role in tissue senescence and damage repair by both accelerating tissue senescence and promoting damaged tissue repair [20, 29]. Senescent cells, although in a state of cell cycle arrest, can influence neighboring cells and tissues by secreting inflammatory factors in order to remain metabolically active; therefore, SASP has been implicated as a trigger for chronic inflammation, oxidative stress, and reduced nitric oxide bioavailability [30]. In summary, the biological effects of cellular senescence are two-sided; short-term acute cellular senescence facilitates cardiac development, embryonic angiogenesis, wound healing, and tissue repair, whereas long-term chronic accumulation of senescent cells leads to tissue dysfunction and promotes tumorigenesis, and in particular contributes to the development of age-related cardiovascular disease.

#### The dual roles of cellular senescence in cardiovascular diseases

Given the complex and dual biological effects that cellular senescence demonstrates across various physiological and pathological processes, it also plays a significant dual role in the specific domain of cardiovascular disease. The research conducted by Anna Walaszczyk et al. demonstrates that the clearance of senescent cells can improve myocardial remodeling and diastolic function, as well as enhance overall survival rates following MI [31]. Furthermore, conventional drug therapies that improve cardiac function after MI also reduce the expression of senescence markers in cardiac tissue [32]. In contrast, a multitude of research endeavors have put forward the notion that cell senescence exerts a positive influence after MI [33, 34]. Notably, a reduction in the synthesis of SASP components is correlated with an exacerbation of systolic dysfunction and an augmentation of cardiac fibrosis during the post-MI period. Such a correlation implies that the SASP components located in the peri-infarct area manifest antifibrotic and cardioprotective properties [35]. Growing evidence demonstrates that cellular senescence plays a pivotal role in vascular pathologies such as atherosclerosis, with recent comprehensive reviews summarizing the underlying mechanisms [36–39]. Cellular senescence exerts anti-atherosclerotic effects by restricting the proliferation of VSMCs and macrophages, suppressing pro-inflammatory cytokine secretion, and stabilizing plaques through p16/p53/p21/ARF-mediated mechanisms, thereby inhibiting plaque formation and reducing vulnerability [40–42]. Interestingly, a study using an endothelial cell (EC)-specific progeroid mouse model demonstrated that EC senescence enhances NF-κB signaling pathway-mediated regulation of VCAM-1 through epigenetic modifications, leading to exaggerated inflammatory responses in the vascular endothelium and increased monocyte adhesion, thereby accelerating atherosclerosis progression [43]. Recent research has indicated that cardiac IR-induced senescent cardiomyocytes secrete SASP, which in turn activates maladaptive cardiac remodeling processes such as cellular hypertrophy, inflammation, fibrosis, and a reduction in regenerative capacity, eventually leading to chronic cardiac fibrosis [35, 44]. Conversely, studies have shown that IR-induced cardiomyocyte senescence can play a cardioprotective role by triggering cardiac fibroblast senescence, specifically by promoting cardiomyocyte proliferation and inhibiting the progression of fibrosis, which in turn promotes regeneration of neonatal heart [45]. In summary, Cellular senescence exhibits a significant dual role in CVDs. On one hand, clearing senescent cells or reducing their biomarker levels improves post-MI cardiac remodeling and function while exerting anti-atherosclerotic effects by inhibiting aberrant proliferation of vascular lesion cells. On the other hand, under specific pathological conditions, senescent cells confer cardioprotection through the anti-fibrotic effects of SASP components or by promoting cardiomyocyte proliferation; however, their excessive activation can also induce chronic fibrosis and maladaptive remodeling.

### Mechanisms regulating cellular senescence

**Fig. 2:**
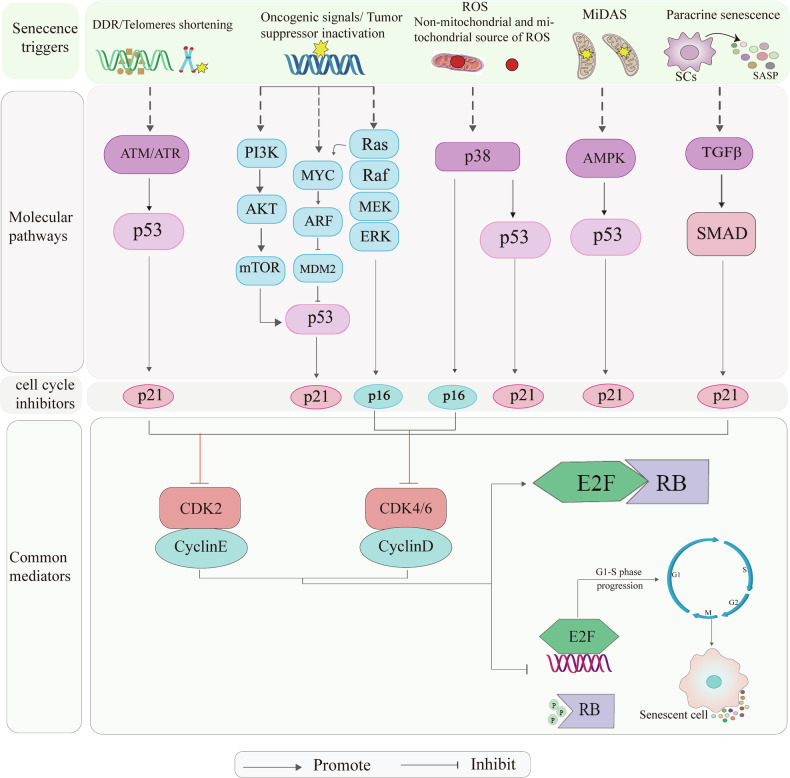
Mechanisms regulating cellular senescence. Senecence triggers include DDR/Telomeres shortening; Oncogenic signals/ Tumor suppressor inactivation; ROS(Non-mitochondrial and mitochondrial source of ROS); MiDAS-Dysfunctional mitochondria; Paracrine senescence. These triggers activate cell cycle protein kinase inhibitors p16INK4A and p21Cip1 by modulating their respective signaling pathways. Elevated expression of these inhibitors leads to the inhibition of cell cycle protein-dependent kinases CDK2 and CDK4/6. Consequently, this inhibition promotes the continuous association of RB with the transcription factor E2F, thereby maintaining RB in a hypophosphorylated state. This prevents E2F from exerting its regulatory role in the cell cycle, ultimately causing cell cycle arrest at the G1 phase.

Cellular senescence is a complex process involving multiple molecular mechanisms. Briefly summarized below are the key molecular mechanisms involved. The normal cell cycle is regulated by a series of functional proteins. Cyclins are a class of proteins closely associated with the cell cycle, whose expression levels fluctuate in tandem with the stages of the cell cycle. They play a regulatory role in the cell cycle progression by binding to and activating specific protein kinases, such as p16. Notably, p16 directly interacts with CDK4/6, inhibiting their activities and exerting regulatory effects on the cell cycle [19]. The senescence-associated marker, p21, plays a crucial role in the normal cell cycle by inhibiting the activity of various cyclin-dependent kinases (CDKs). When cellular senescence is induced by different stimuli, the expression of p21 is consistently up-regulated. Its primary regulatory mechanism involves direct activation by p53, although other pathways, such as TGF-β, can also mediate this process [46–49]. During the normal cell cycle, the unbound retinoblastoma protein (Rb) is phosphorylated by CDK4/6 or CDK2 proteins. This phosphorylated Rb then activates and releases the transcription factor E2F, which serves as a key regulator in promoting progression from the G1 phase to the S phase. Conversely, when the cell cycle protein kinase inhibitors p16INK4A and p21Cip1 are highly expressed, they inhibit Rb phosphorylation mediated by CDK4/6 or CDK2, resulting in continuous binding between unbound Rb and E2F. As a consequence, E2F is unable to fulfill its role in regulating the cell cycle, leading to cell cycle arrest at the G1 phase [50–52]. During each round of DNA replication, telomeres gradually shorten as cell division progresses. Eventually, they reach a critical length that inhibits further replication, leading to the cessation of cell division. Additionally, shorter, uncapped telomeres elicit a DNA damage response (DDR), which subsequently induces cellular senescence. Sustained DNA damage triggers a DNA damage response (DDR), where double-stranded DNA breaks (DSBs) act as robust stimuli activating the DDR mechanism. Failure to promptly address this mechanism leads to cellular senescence. Moreover, DSBs facilitate the recruitment and binding of ATM kinases to DNA damage sites, ultimately inducing cell-cycle arrest and senescence through the activation of the p53/p21 and p16/pRb pathways [53, 54]. Over-activation of oncogenes, such as RAS, or inactivation of tumor suppressor genes, like PTEN, can also initiate DDR, resulting in cellular senescence known as oncogene-induced senescence (OIS) [55]. Certain components of SASP, which are released by senescent cells, also play a role in initiating cellular senescence. Forman and Zhang proposed that endogenous and exogenous stressors, originating from both mitochondrial and non-mitochondrial sources, can stimulate the production of reactive oxygen species [56]. During the initiation and progression of aging, ROS exerts a signaling function by directly instigating oxidative modifications in subcellular structures such as nucleic acids and enzymes. This activation subsequently leads to oncogene activation, DNA damage, and downregulation of telomerase activity [16]. Mitochondrial dysfunction is closely associated with the generation of reactive oxygen species, and both contribute to elevated oxidative damage, encompassing sulfhydryl oxidation, lipid peroxidation, and mutations in mitochondrial DNA [57, 58] (Fig. 2). Moreover, aging is accompanied by various epigenetic changes such as DNA methylation, histone acetylation, aberrant chromatin remodeling, and dysfunction of non-coding RNA [16]. In summary, the molecular mechanisms of cellular senescence are complex and diverse, primarily involving key signaling pathways and molecular events such as Replicative Senescence, stress-induced premature senescence (SIPS), OIS, SASP, epigenetic regulation, and metabolic alterations. However, the regulatory mechanisms of cellular senescence in CVDs involve multiple molecular events, primarily encompassing core pathways such as telomere shortening, mitochondrial dysfunction, ROS accumulation, genomic instability, epigenetic dysregulation, and protein misfolding. Within the molecular network of cardiac dysfunction, key signaling molecules—including the tumor suppressor p53, p38 mitogen-activated protein kinase (p38MAPK), p21^WAF1/Cip1^-activated kinases (PAKs), and the mammalian target of rapamycin (mTOR)—along with their mediated cascades, constitute the central hubs regulating cellular senescence and cardiac pathological processes. These details have been systematically elucidated in the review by Konstantinos Evangelou et al. Based on the aforementioned regulatory mechanisms, the subsequent sections will systematically elucidate the dynamic roles of cellular senescence in various CVDs, as well as its theoretical foundations as a therapeutic target.

## Cellular senescence and cardiovascular disease

### The role of cellular senescence in hypertension

Hypertension is a significant global health issue that is highly prevalent among the elderly population [59]. It has been described as a condition of premature vascular aging, relative to actual chronological age. Many factors that contribute to the deterioration of vascular function as we age are accelerated in hypertension. Abeywardena et al., Guzik and Touyz showed that, in hypertension, however, the decline in vascular function and aged phenotype are premature in their onset and particularly pronounced [60, 61]. The specific mechanism of the relationship between cellular senescence and hypertension involves multiple levels, including molecular, cellular, and organizational levels. As people age and develop hypertension, the production of reactive oxygen species (ROS) increases, which may be a key link connecting the role of endothelin-1 and p66shc in vascular aging and hypertension [62]. The shortening of arterial telomeres can result in telomere structure destabilization and activation of the P53/P21 signaling pathway, which leads to cellular senescence and contributing to the development of hypertension [63]. Various types of hypertension and organs in hypertensive patients demonstrate cellular senescence, a characteristic feature of end-stage organ damage. A wide range of pro-hypertensive stimuli can induce vascular cell senescence, which further promotes a senescent phenotype and contributes to persistent hypertension. Additionally, autophagy is implicated in hypertension, as vascular cells experience prolonged autocrine, paracrine, and endocrine stress and damage. Three potential pro-senescence mechanisms, namely autophagy dysregulation, endoplasmic reticulum stress, and proteotoxicity, along with telomere depletion, are closely associated with the field of hypertension [64]. Hypertension is characterized by endothelial dysfunction and increased arterial stiffness. Prolonged senescence of smooth muscle cells and endothelial cells elicits an elevation in the levels of Ang II and endothelin 1, subsequently resulting in hypertension [65, 66]. Jens H. Westhoff et al. demonstrated that elevated blood pressure induces p16 expression in rat kidneys, hearts, and human kidneys [67]. The increase in heart and kidney weight in hypertensive rats is associated with specific gene loci, which are related to genetic regulation during cell proliferation and apoptosis [68]. Promising evidence supports targeted interventions in vascular senescence as a novel approach to controlling blood pressure and enhancing vascular function in hypertensive patients. While the exact cause of aging-associated hypertension remains unclear, a substantial body of evidence indicates that endothelial cell senescence plays a role in hypertension. This occurs through the narrowing of small arterioles and decreased capillary bed volume, resulting in increased peripheral resistance. While the exact cause of aging-associated hypertension remains unclear, a substantial body of evidence indicates that endothelial cell senescence plays a role in hypertension. This occurs through the narrowing of small arterioles and decreased capillary bed volume, resulting in increased peripheral resistance. In conclusion, there is a lack of comprehensive understanding regarding the specific regulatory mechanisms underlying cellular senescence in hypertension. Furthermore, further research is required to investigate the role of cellular senescence as an independent mechanism in hypertension.

#### Effects of targeted cellular senescence-related therapies on hypertension

Multiple mechanisms, including oxidative stress, telomere length and activity alterations, vascular endothelial cell senescence, and genetic factors, contribute to the complex and diverse relationship between cellular senescence and hypertension. Consequently, targeting and regulating these aforementioned mechanisms can potentially delay the onset and progression of hypertension. Anti-aging drugs such as Rapamycin, which partially alleviates hypertension by inhibiting the mTOR signaling pathway and improving arterial structure and function, and diastolic function [69]. Anti-hypertensive therapy (hydrochlorothiazide, hydralazine, and reserpine), losartan, and spironolactone all attenuate p16 expression [67], and the use of ACEI and ARB antihypertensive drugs has been shown to reduce levels of oxidative stress. Nicotinamide adenine dinucleotide (NAD^+^) is an essential cofactor in all living cells, playing a crucial role in fundamental biological processes. NAD^+^ levels decline with aging [70, 71]. Recent studies have demonstrated that the administration of NAD+ improves endothelium-mediated vasodilation and reduces arterial stiffness in aged mice [72]. Supplementation with the NAD^+^ precursor nicotinamide ribose has been found to reduce arterial stiffness in healthy older adults [73]. Moreover, Nicotinamide phosphoribosyltransferase (NAMPT) is a potentially cardioprotective adipocytokine that plays an important role in DNA damage repair and prevention of premature aging in VSMCs, as well as a key enzyme in the regulation of NAD^+^ biosynthesis and SIRT1 activity [74, 75]. Overexpression of NAMPT partially inhibited Ang II-induced elevation of ROS levels by regulating the concentrations of SIRT1 and NAD^+^, thereby alleviating Ang II-induced hypertension [76]. Therefore, modulating NAD^+^ levels represents a potential anti-aging strategy with therapeutic implications for hypertension and other age-related diseases. Importantly, Ryohei et al. reported that resveratrol suppresses the expression of AT1R in the mouse aorta by activating SIRT1 and ameliorates Ang II-induced hypertension. Meanwhile, overexpression of SIRT1 reduces the expression of AT1R, while SIRT1 inhibitor (nicotinamide) reverses this effect [77]. The decreased expression of SIRT1 in the paraventricular nucleus of hypothalamus (PVN) of hypertension leads to increases of NF-κB p65 activity, inflammasome activation, thereby enhancing sympathetic nerve activity and aggravating hypertension [78]. In summary, the association mechanisms between cellular senescence and hypertension are complex. Through various targeted interventions (e.g., the application of anti-aging drugs, regulation of NAD^+^ levels, and the use of specific substances to modulate SIRT1 activity), positive impacts can be exerted on the occurrence and development of hypertension, providing new ideas and potential strategies for the prevention and treatment of hypertension and other aging-related diseases (Table 1).

**Table 1:** Impact of targeted cellular senescence-related therapies on cardiovascular disease.

Cardiovascular disease	Drug targets or mechanisms	Effect	Refs
Hypertension	The shortening of arterial telomeres	Promote	[49]
Hypertension	Activation of the P53/P21 signaling pathway	Promote	[49]
Hypertension	Rapamycin inhibiting the mTOR signaling pathway	Partially alleviates	[55]
Atherosclerosis	Inhibition of glutaminase	Alleviate	[93]
Atherosclerosis	Genetic ablation of gpnmb-positive cells	Alleviate	[95]
Atherosclerosis	D & Q can reduce the expression level of aging markers	Alleviate	[95]
Atherosclerosis	ABT-263 selectively eliminate senescent cells	Alleviate	[96]
Myocardial infarction	Venetoclax attenuating SASP-associated inflammatory responses	Alleviate	[99]
Myocardial infarction	SIRT1 boost the anti-apoptotic and angiogenic capacities of senescent mesenchymal stem cells	Alleviate	[111]
Heart failure	SIRT6 preventing the differentiation of fibroblasts towards myofibroblast differentiation	Alleviate	[80]
Heart failure	SIRT1 enhances PGC-1αactivity	Alleviate	[126]
Heart failure	ABT-263 improve left ventricular contractile function, reduce myocardial fibrosis and hypertrophy	Alleviate	[95]
Arrhythmias	Quercetin inhibiting TGF-β/Smads pathway	Alleviate	[150]

### The role of cellular senescence in atherosclerosis

Atherosclerosis is a chronic inflammatory vascular disease associated with aging, where cellular senescence plays a significant role as a major risk factor. It is characterized by the presence of lipid-rich plaques in the arterial wall [79]. Endothelial dysfunction, marked by enhanced inflammation, oxidative stress, persistent DNA damage, increased expression of cell cycle-blocking proteins, and cellular senescence, is one of the main contributors to the development of atherosclerosis [80]. Plaque formation and expansion involve smooth muscle cell proliferation and reduced levels of endothelial-type nitric oxide synthase. These events can result in telomere shortening and oxidative stress, respectively. Given the intricate signaling interactions among smooth muscle cells, endothelial cells, and immune cells recruited to the plaque, these findings suggest that cellular senescence could be involved in various stages of atherogenesis [81–83]. In conclusion, cellular senescence plays a significant role in atherosclerosis by mediating endothelial cell dysfunction and stabilizing atherosclerotic plaques. Gaining a comprehensive understanding of strategies to reverse cellular senescence, repair or replace senescent cells, and knowledge of the interactions between cellular senescence, inflammatory responses, cholesterol metabolism, and other relevant factors are vital. This understanding can contribute to the development of more effective treatments and preventive measures for atherosclerosis.

#### Cellular senescence mediates vascular endothelial dysfunction

Endothelial cells are essential for maintaining the structural integrity and homeostasis of blood vessels [84]. Previous studies have demonstrated that endothelial cell senescence is the initiating link in the development of atherosclerosis [85]. However, senescent endothelial cells impair both endothelial integrity and permeability, consequently facilitating the accumulation of oxidized low-density lipoprotein (ox-LDL). This accumulation further stimulates the intracellular production of reactive oxygen species (ROS) and induces mitochondrial dysfunction, thereby promoting the progression of atherosclerosis [86–89]. In cardiovascular disease studies, the shortening of telomeres in leukocytes within atherosclerotic coronary arteries has been identified as a significant characteristic of cellular senescence. This state of cellular senescence then triggers endothelial hyperinflammation through epigenetic modifications. Consequently, the process of atherosclerosis is accelerated [43]. Previous studies have shown that inhibiting telomere function induces senescence in human aortic endothelial cells (HAECs), resulting in increased expression of intercellular adhesion molecule (ICAM)-1and a decrease in activity of endothelial nitric oxide synthase (eNOS). This phenomenon has been linked to the development and progression of atherosclerosis [39]. During the aging process, the extracellular matrix of the inner wall of the blood vessels undergoes a process of hardening, leading to an increase in the permeability of endothelial cells. This increased permeability further facilitates the extravasation of leukocytes, which plays a vital role in the formation of atherosclerotic plaques [86]. Functionally abnormal senescent endothelial cells additionally exhibit reduced expression of endothelial nitric oxide synthase while showing an increased expression of pro-inflammatory molecules and adhesion molecules. These changes, in turn, trigger vascular inflammation [90–92]. Senescence of endothelial cells leads to adverse effects, including dysregulation of blood flow and barrier dysfunction, hindering the ability of the vascular lumen to repair itself due to the inability of senescent cells to proliferate [93]. Secondary senescence, induced by IL-1β, in human endothelial cells and/or vascular smooth muscle cells, may be a mechanism that leads to the accumulation of senescent cells. This mechanism potentially contributes to the development of atherosclerosis and its associated complications. IL-1β can additionally contribute to endothelial cellular senescence in human EC by up-regulating CUX1 expression or down-regulating SATB2 expression [94]. Endothelial cell senescence leading to atherosclerosis is promoted by SIRT6 deficiency or miR-217 overexpression. FOXM1, a key transcription factor for cell cycle progression and senescence, plays a critical role. FOXM1 overexpression ameliorates endothelial cell senescence induced by SIRT6 deficiency, thereby alleviating atherosclerosis [95, 96]. In summary, given the pivotal driving role of endothelial cell senescence in atherosclerosis, the development of anti-aging therapies targeting telomere function, miROS, or the miR-217/SIRT6 regulatory axis may provide novel therapeutic targets for improving vascular repair capacity and inhibiting plaque progression.

#### The role of cellular senescence in plaque formation and plaque stability

**Fig. 3:**
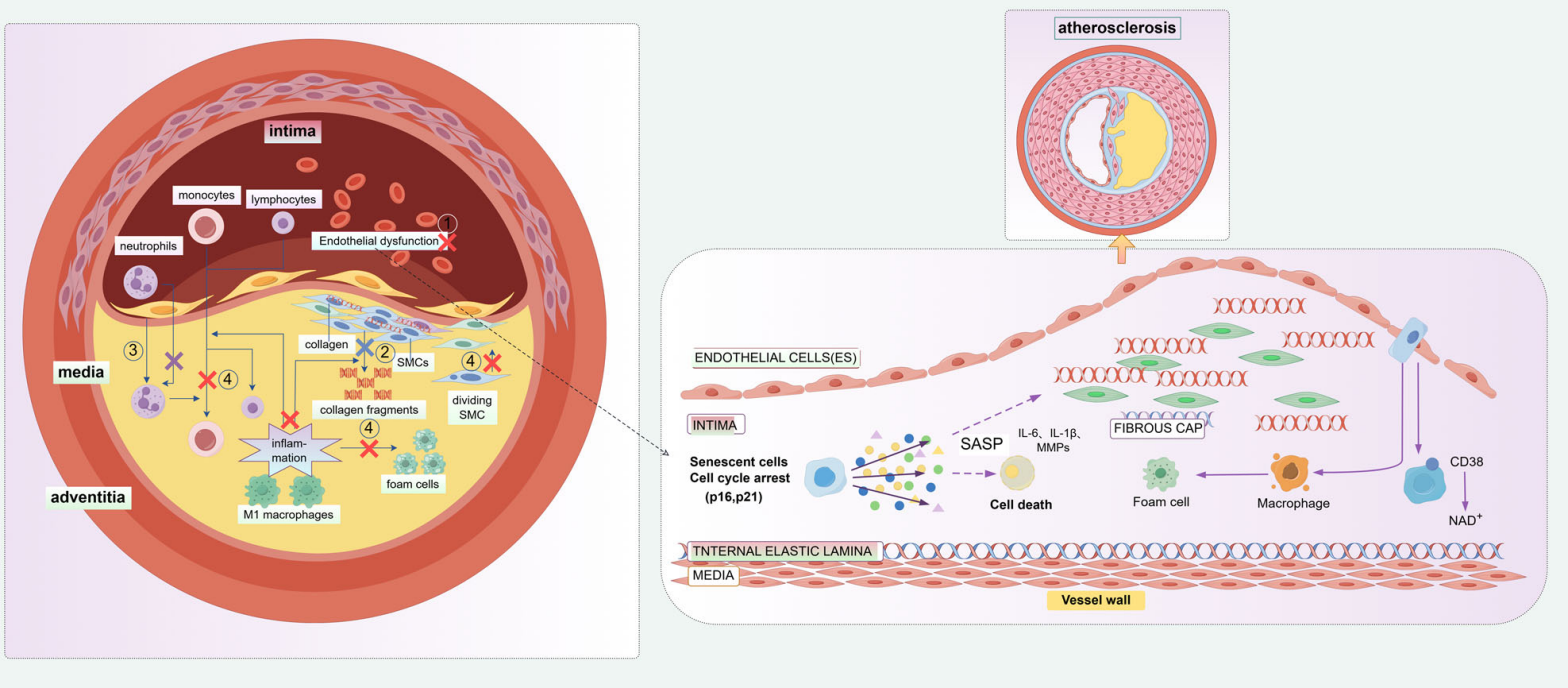
Schematic diagram of the mechanism by which cellular senescence functions in the progression of atherosclerosis. The left side shows a cross-section of an arterial vessel. In the intima, there is infiltration of immune cells such as monocytes, lymphocytes, and neutrophils, along with endothelial dysfunction. Smooth muscle cells (SMCs) exhibit abnormal proliferation and secrete collagen and fragments. Additionally, there is an inflammatory response and accumulation of M1 macrophages. The right side presents a local magnification and further mechanistic details. Senescent endothelial cells undergo cell cycle arrest (Cell cycle arrest, p16,p21) and secrete the senescence-associated secretory phenotype (SASP, including IL-6, IL-1β, MMPs), leading to cell death, foam cell formation, macrophage activation, It also involves structural changes in the fibrous cap (FIBROUS CAP) and pathological alterations in various layers of the vessel wall (including the intima, internal elastic lamina, and media), ultimately promoting the development and progression of atherosclerosis.

Atherosclerotic plaques are lesions that result from a combination of endothelial damage, excessive proliferation of smooth muscle cells, and an overproduction of extracellular matrix [97] (Fig. 3). Atherosclerosis contributes to atherosclerotic plaque formation through inflammatory infiltration, impaired endothelial function, accumulation of low-density lipoprotein cholesterol, and proliferation and migration of vascular smooth muscle cells [98]. Plaque formation initially originates from senescent endothelial cells that mediate the invasion of circulating monocytes into the vascular wall through self-secreted senescence-associated secretory phenotype and surface receptors [99]. Additionally, senescent endothelial cells are prone to apoptosis, resulting in the “leakage” of the endothelial layer. This phenomenon facilitates the permeation of oxidized LDL into the vessel wall [100, 101]. However, senescent endothelial cells are incapable of effectively performing their functions, which include secreting NO to inhibit smooth muscle cell proliferation and prevent lipid peroxidation [102]. This can result in the early thickening of the endothelium, which is a crucial risk factor for atherosclerosis. Secondly, the progression of plaques may be facilitated by chemokines found in the SASP, including certain cytokines like MCP1 and interleukins, which are known to promote atherosclerosis [103]. Lastly, with the progression of cellular aging, plaques undergo destabilization and become more susceptible to rupture. This leads to the formation of vulnerable plaques, which significantly increase the risk of acute complications such as myocardial infarction and cerebral infarction [104]. Grootaert MOJ et al. showed that the association between the senescence of vascular smooth muscle cells and the advancement and instability of atherosclerotic plaques. Additionally, it has been observed that the absence of VSMCs induces cell death, resulting in the thinning of the fibrous cap and facilitating the development of necrotic core formation and calcification [105]. Furthermore, senescent VSMCs demonstrate an upregulation of chemokines such as CCL2, adhesion molecules including ICAM-1, and innate immune receptors. These properties contribute to the creation of an inflammatory environment, which subsequently enhances the migration of inflammatory cells. Consequently, the senescence of VSMCs not only accelerates the progression of atherosclerosis but also hampers the repair process of plaques [106]. Advanced stages of atherosclerotic lesions are found to contain senescent cells. These cells promote the formation of atherosclerosis by upregulating matrix metalloproteinases and aggravating vascular inflammation, causing plaque destabilization [107, 108]. As a result of limited proliferation of senescent cells in atherosclerotic plaques, there is increased expression of the inhibitory factor of the cell cycle, p16^INK4A^ protein. This results in enhanced senescence-associated β-galactosidase activity and the establishment of SASP, leading to the secretion of multiple inflammatory cytokines, chemokines, and matrix-degrading proteases. Reducing the expression of the cell cycle inhibitory factor p16^INK4A^ protein slows atherogenesis and promotes plaque stability [109]. In mice, SIRT6 retards the aging of VSMCs. Additionally, activation of sirtuin protects telomeres from damage and decreases the production of inflammatory cytokines, increasing stability in atherosclerotic plaques [110]. In conclusion, cellular senescence leads to abnormalities in cellular function and metabolism, decreasing the vascular wall’s ability to repair and remove cholesterol. This promotes the formation of atherosclerotic plaques comprising vascular endothelial cells, smooth muscle cells, and macrophages. Furthermore, cellular senescence induces abnormal cellular function, apoptosis, and an increase in the inflammatory response within the plaques. Consequently, this diminishes plaque stability. In summary, cellular senescence is involved in the regulation of plaque formation and stability in atherosclerosis through multiple mechanisms. Further studies on the mechanisms of cellular senescence may help to develop new therapeutic strategies to attenuate the development and progression of atherosclerosis.

#### Effects of targeted cellular senescence-associated therapy on atherosclerosis

A study showed that by eliminating Senescent cells(SNCs) as they accumulate during atherogenesis, hypercholesteremic Ldlr^−/−^ mice develop fewer lesions and lesions that do form are smaller and more stable. SNCs do so by antagonizing IGF-1 through the secretion of insulin-like growth factor-binding protein 3 (IGFBP-3). These data indicate that the intermittent use of senolytic agents or IGFBP-3 inhibition in combination with lipid-lowering drugs may provide therapeutic benefit in atherosclerosis [111]. Chronic clearance of senescent cells improves established vascular phenotypes associated with aging and chronic hypercholesterolemia, and may be a viable therapeutic intervention to reduce morbidity and mortality from cardiovascular diseases [112]. Johmura et al. results suggest that glutaminase 1 (GLS1) is an essential gene for the survival of human senescent cells, and that inhibition of glutaminase reduces atherosclerotic plaque formation [113]. Suda M et al. study showed that glycoprotein nonmetastatic melanoma protein B (GPNMB) expression was upregulated in vascular endothelial cells and/or leukocytes of patients and mice with atherosclerosis. Genetic ablation of Gpnmb-positive cells attenuated senescence in adipose tissue and improved systemic metabolic abnormalities in mice fed a high-fat diet, and reduced atherosclerotic burden in apolipoprotein E knockout mice on a high-fat diet [114]. The combination of D & Q, another anti-aging drug, can reduce the expression level of aging markers in hypercholesterolemia mice, improve vascular vasomotion, and inhibit arterial calcification [115]. Bcl-2 inhibitors such as ABT-263 have been proven to selectively eliminate senescent cells during atherosclerosis, thereby delaying the progression of the disease [107]. Furthermore, SIRT1, as an anti-aging agent, has been extensively studied in the context of EC senescence and atherosclerosis. The earliest experimental evidence has shown that chemical or siRNA inhibition of SIRT1 leads to senescence in HUVECs via enhancing the acetylation and activity of p53 [116]. Overexpression of SIRT1 prevents H2O2-induced EC senescence and downregulation of eNOS [116]. Subsequent studies demonstrated that overexpression of SIRT1 in the endothelium can improve vascular stiffness and attenuate the development of atherosclerosis, probably by activating eNOS and promoting NO production and preventing EC senescence [116–118]. Interestingly, global Sirt1 heterozygous KO enhances endothelial inflammation, without affecting eNOS activity, in atherosclerotic Apoe^−/−^ mice [119]. SIRT1 in hematopoietic cells also prevents foam cell formation and reduces atherosclerosis [120]. Previous studies have demonstrated that SIRT1 can activate FOXO3a through deacetylation, thereby upregulating the expression of the MnSOD gene and enhancing its transcriptional activity. Through the SIRT1/FOXO3a/MnSOD axis, SIRT1 inhibits oxidative stress and the generation of reactive oxygen species (ROS), protects vascular endothelium, and improves cardiac function [121]. Zhang et al. discovered that upregulating the expression of SIRT1 can inhibit oxidative stress and inflammatory responses by regulating the AMPK/SIRT1/NF-κB signaling pathway, thereby improving the progression of atherosclerosis [122]. Downregulation of SIRT1 causes OVX-induced arterial senescence and atherosclerosis in ApoE-KO mice. Administration of estrogen or SERM enables OVX mice to restore these alterations by SIRT1 induction [123]. In conclusion, the above studies provide theoretical studies for targeting cellular senescence-related therapies for mitigating the development of atherosclerosis (Table 1).

### The role of cellular senescence in myocardial infarction

Myocardial infarction (MI) is a leading cause of death in elderly individuals and results in extensive structural changes in the heart, characterized by hypoxic and dystrophic pathology of cardiomyocytes due to coronary artery occlusion. Although cardiomyocytes undergo terminal differentiation, senescent cardiomyocytes have been observed in a variety of cardiovascular diseases, including MI [124]. The phenomenon of cellular senescence increases with age and is associated with a decline in the function of tissue-specific stem/progenitor cells. In the elderly population, over 50% of cardiac progenitor cells exist in a senescent state, rendering them incapable of replication, differentiation, regeneration, or reinstating cardiac function [125]. This suggests that cellular senescence may affect cardiac remodeling after myocardial infarction by reducing the function of cardiac progenitor cells. Findings from previous studies suggest that myocardial infarction or hypoxia causes fibroblast senescence, elevated cytokine expression, accumulation of collagen, and impairment of cardiac fibrosis, resulting in an elevated risk of cardiac rupture. Moreover, these outcomes are linked to the activation of the p53 signaling pathway. Therefore, inhibiting p53 activity could be a viable target in the treatment of reparative cardiac fibrosis and prevention of myocardial rupture following myocardial infarction [126]. Myocardial infarction commonly leads to extensive loss of cardiomyocytes, and ischemic injury induces DNA damage, oxidative stress, and mitochondrial dysfunction, making cardiomyocytes susceptible to undergo senescence. On the other hand, targeted inhibition of cardiomyocyte senescence enhances cardiac function, decreases the size of the scar, and mitigates senescence of interstitial cells after ischemia-reperfusion injury [127]. SASP Factors in Cardiomyocyte Senescence Additionally Induce Senescence in neighboring cells and contribute to Fibrotic tissue formation and scar formation [127]. Furthermore, p16 plays a key role in regulating cardiomyocyte senescence, and p16 expression in cardiomyocytes is increased with age and in response to myocardial infarction [44, 128]. When p16 is inactivated, it suppresses p21 expression, and knockdown of p16 also has the potential to reduce activation of the p53/p21 pathway by reducing oxidative stress, thereby preventing DNA damage [127]. Mitochondria serve as the primary source of ATP in cardiomyocytes and participate in division and fusion processes to maintain their function. Disruption of the balance between mitochondrial division and fusion is responsible for mediating cellular senescence. Furthermore, ischemic injury induces senescence of cardiomyocytes through activation of mitochondrial fission, resulting in dysfunction of the heart [129, 130]. For instance, under hypoxic stress, interactions between Drp1 and filamin A have been shown to cause mitochondrial hypersegmentation, resulting in cardiomyocyte senescence in a mouse infarction model. Senescent cardiomyocytes contribute to cardiac injury by impairing the function of cells other than cardiomyocytes, such as endothelial cells and fibroblasts, by secreting certain factors as part of the senescence-associated secretory phenotype [131]. A study demonstrated that Hemin-MSC-EXO exhibited high levels of miR-183-5p expression, partly through the regulation of the HMGB1/ERK pathway. This expression was found to be effective in inhibiting ischemia-induced cardiomyocyte senescence and enhancing the cardioprotection through the regulation of mitochondrial division [130]. In the context of myocardial infarction, SASP negatively impacts cardiac structure and function through their pro-inflammatory and ability to damage surrounding tissues. For example, certain proteins in SASP, such as GDF15, CST6, and IGFBP2, correlate significantly with inflammatory markers, renal function, and hematological profiles [132]. This suggests that SASP may further exacerbate cardiac injury after myocardial infarction by affecting the inflammatory response and tissue repair processes. In summary, cellular senescence plays a key role in the development of myocardial infarction and subsequent cardiac remodeling, and affects cardiac function and structure through multiple mechanisms. In-depth investigation of cellular senescence-related pathways and intervention targets is of great clinical significance for improving the prognosis of myocardial infarction and mitigating cardiac injury.

#### Effects of targeted cellular senescence-associated therapy on myocardial infarction

Neovascularization after myocardial infarction is important for the recovery and maintenance of cardiac function [133, 134]. Endothelial cells are the main cell type that initiate and maintain the angiogenic process, mediating vascular endothelial growth factor, the fibroblast growth factor, and hypoxia-inducible factor [65]. Endothelial cell aging reduces the expression of vascular endothelial growth factor, resulting in reduced angiogenic capacity [135]. He L, et al. showed that the use of venetoclax to remove senescent cardiac cells following cardiac ischemia-reperfusion has been found to attenuating SASP-associated inflammatory responses, promoting angiogenesis, reducing scar formation, and improving cardiac function in preclinical studies [99]. Gorski et al. provided clarification on the role of SIRT1 as a regulator, demonstrating its ability to enhance cellular function in aging conditions by reducing apoptosis in cardiomyocytes. By promoting the deacetylation of SERCA2a, SIRT1 restores its activity and consequently enhances cardiac function. Furthermore, SIRT1 has the potential to boost the anti-apoptotic and angiogenic capacities of senescent mesenchymal stem cells (MSCs), thus augmenting their therapeutic efficacy against myocardial infarction [136]. This suggests that by targeting SASP-related pathways or utilizing modulators such as SIRT1 may provide new therapeutic strategies for myocardial infarction. Lee et al. found that biodegradable poly(lactic-co-glycolic acid) nanoparticle-based local delivery of a senolytic drug (ABT263-PLGA) successfully eliminated SISCs in the myocardial ischemia-reperfusion injured rat hearts; the treatment helped restore cardiac function, ameliorated inflammatory responses, and attenuated adverse remodeling [137]. Based on the above studies on factors associated with MI and aging, the related signaling pathway p53/p21 also plays an important role in MI, which is summarized in the following Table 2. In conclusion, the current results are limited to the animal experimental stage and still have a distance to clinical safe application. We still need to further explore the specific mechanisms of targeted cell senescence-related treatment in myocardial repair and angiogenesis after myocardial infarction, in order to more effectively achieve clinical translation and bring benefits to patients in the future (Table 1).

**Table 2:** Effect of modulation of p53/p21 signaling pathway on myocardial infarction.

Disease	Experimental subject	Senescence pathway	Drug targets /mechanisms	Effects	Refs
Myocardial infarction	CMs / CFs C57BL/6 mice	p53/p21 pathway	Via a GATA4-CCN1-fibrosis pathway	Negative p16 ↑ , p53 ↑ , p21 ↑ , SASP ↑ , SA-β-gal↑	[138]
Myocardial infarction	NRCMs Rats/mice	p53/p21 pathway	Drp1 mediated mitochondrial fission-associated myocardial senescence	Negative p53 ↑, SA-β-gal ↑	[139]
Myocardial infarction	CMs C57BL/6 mice	p53/p21 pathway	HO-1 inhibited cardiomyocyte senescence of aged heart	Negative p53 ↑, p16 ↑, SA-β-gal ↑, SASP↑	[140]
Myocardial infarction	C57BL/6N mice	p53/p21 pathway	CCN1 induces a DNA damage response and p53 activation, which activate the p16/Rb pathways to induce senescence	Negative SA-β-gal ↑, p16 ↑, p21 ↑, p53 ↑	[141]
Myocardial infarction	C57BL/6J mice NRCMs / CFs	p53/p21 pathway	Sirt1 controls p53 and AC-p53, and stops aging markers	Negative p21 ↑, p53 ↑, p19 ↑, SA-β-gal ↑, SIRT1↓	[142]

*CMs* cardiac myocytes, *NRCMs* Neonatal rat cardiomyocytes, *CFs* cardiac fibroblasts.

### The role of cellular senescence in heart failure

Heart failure represents structural and/or functional abnormalities of the heart resulting from systolic and/or diastolic dysfunction. This condition represents the final stage in the development of cardiovascular disease. Numerous studies have demonstrated a close relationship between cellular senescence and heart failure [138]. As the body ages, it progressively impacts the heart, resulting in a reduction in cardiomyocyte count. Meanwhile, the surviving cardiomyocytes undergo remodeling and contribute to cardiac fibrosis. This process ultimately leads to a significant decrease in both pump function and contractile reserve [139]. Previous studies have demonstrated that under conditions of left ventricular pressure overload, sympathetic signaling is activated. This leads to the activation of p53 signaling within cardiac microvascular endothelial cells, subsequently resulting in an increase in the expression of p53 and ICAM-1 within these cells. Consequently, macrophage infiltration and cardiac inflammation are promoted. This process may ultimately contribute to the development of heart failure [140]. Numerous experimental studies have demonstrated that endothelial dysfunction exists in senescence-accelerated mice (SAMP mice), and a diet high in salt and fat accelerates senescence in endothelial cells. This, in turn, induces endothelial inflammation, resulting in HFpEF characterized by diastolic dysfunction, left ventricular hypertrophy, left atrial dilatation, and interstitial fibrosis [141]. At the same time, aging cardiomyocytes also exhibit heart failure, characterized by prolonged diastolic duration, reduced contraction velocity, attenuated beta-adrenergic responsiveness, and elevated myocardial stiffness [142]. In the aging heart, senescent cardiac cells exhibit senescence-associated markers, such as p16, p21, and p53, accompanied by telomere shortening. Biopsies of the heart muscle from patients with heart failure indicate telomere shortening and elevated cellular senescence [143, 144], The above phenomenon demonstrates that cellular senescence and heart failure are closely related and mutually reinforcing.

#### Cellular senescence in heart failure

Cellular senescence can interact with various pathological mechanisms in heart failure, such as mitochondrial dysfunction, autophagy disruption, and the activation of the neurohumoral system [145–147]. Pim1 functions as a conserved serine/threonine protein kinase with various protective effects on mitochondrial function and telomere length. Furthermore, research has found that Pim1-deficient mice develop heart failure and exhibit elevated levels of markers associated with aging, including p16, p53, and SA-β-gal. Additionally, deterioration of mitochondrial structure and function is observed within cardiac tissue [148, 149]. PI3K inhibition induces autophagy, preserves cardiac function, and decreases levels of p16, p21, p53, and select SASP components in cardiac tissues of aged mice, suggesting a relationship between autophagy and aging during heart failure [150]. Cardiac pathological conditions like myocardial infarction and hypertension result in a gradual accumulation of senescent cells in cardiac tissue. These senescent cells contribute to the development of heart failure by increased fibrosis, inflammation, and oxidative stress, among other responses [115]. Myeloid-derived suppressor cells (MDSCs) derived from the myeloid lineage accumulate within aging tissues. These cells have been shown to be linked to various aging-related diseases [151]. Granulocytic myeloid-derived suppressor cells (G-MDSCs) can potentially inhibit fibroblast senescence and programmed cell death by modulating the FGF2-SOX9 signaling pathway in fibroblasts and induce cell cycle arrest. Additionally, they have the potential to promote cardiac fibrosis and impair cardiac diastolic function, ultimately increasing the risk of heart failure [151]. In addition, cardiomyocyte senescence leads to hypertrophy and fibrosis in the senescent heart, and elimination of these senescent cells may promote cardiomyocyte regeneration [145]. Overall, cellular senescence is an important pathological mechanism underlying heart failure, exacerbating the reduction of myocardial contractility, impaired myocardial diastolic function, and the worsening of cardiovascular disease. Prevention and treatment of cellular senescence in heart failure are crucial measures to protect heart health and prevent the development of cardiovascular diseases. Hence, developing therapeutic methods or drugs to reverse cellular senescence may be a promising research direction.

#### Effects of targeted cellular senescence-associated therapy on heart failure

The targeted cell senescence-related treatment aims to improve heart function by regulating the process of cell senescence. SIRTs are enzymes with anti-aging properties, and many studies have shown that SIRTs play an influential role in processes related to heart failure (HF), such as cardiac hypertrophy, cell death, and oxidative stress. SIRTs are also involved in cardiac remodeling and in HF development [98]. Therefore, during heart failure, SIRT6 is able to exert a protective mechanism in preventing the differentiation of fibroblasts towards myofibroblast differentiation [98]. Inadequate peroxisome proliferator-activated receptor-γ coactivator 1-αactivity triggers mitochondrial dysfunction and heart failure. Nevertheless, SIRT1 enhances PGC-1αactivity through the restoration of metabolic function in the failing myocardium, thus alleviating the condition of heart failure [152]. The Inhibition of PPARα and SIRT1 worsens heart failure by promoting impaired mitochondrial function through the estrogen-related receptor gene expression cascade [153]. G-MDSCs induce cardiac fibrosis by promoting the proliferation of cardiac fibroblasts. Fibrosis associated with cellular senescence is an important pathological factor in Heart Failure with Preserved Ejection Fraction. G-MDSCs serve as a new antifibrotic therapeutic target for HFpEF [151]. Previous studies have shown that the p53 pathway plays a crucial role in the progression from left ventricular hypertrophy to heart failure [140]. Inhibiting either p53 or p16 has been demonstrated to extend the lifespan of various cell types. Thus, inhibiting p53 in endothelial cells holds promise as a new target for treating HFpEF [154]. Ischemic heart disease (ICM), resulting from underlying coronary artery disease, represents the leading cause of heart failure and is a significant contributor to mortality among individuals diagnosed with heart failure. Previous studies have demonstrated that the expression levels of the aging-associated signature (CSA) genes MYC, STAT3, and MAP2K1 were significantly reduced in ischemic heart disease. These three CSAs have the potential to serve as both biomarkers and therapeutic targets for the development of novel therapeutic strategies for heart failure [138]. Because senescent cells generally possess anti-apoptotic properties, anti-apoptotic protein Bcl-2 inhibitors such as ABT-263 have been shown to improve left ventricular contractile function, reduce myocardial fibrosis and hypertrophy, and inhibit heart failure [115]. Hence, targeting cellular senescence-associated therapy may be a novel therapeutic avenue for heart failure with preserved ejection fraction. Further research and exploration are needed to determine whether targeting cellular senescence can be an effective treatment for heart failure (Table 1).

### The role of cellular senescence in arrhythmias

#### Effects of cellular senescence on cardiac electrophysiology

The field of cardiac electrophysiology seeks to comprehend the normal function of electrical activity in the heart. Simultaneously, the atrial and ventricular muscles contract and relax, displaying rhythmic contractions that are reflected in the electrochemical activity of depolarization and repolarization in the heart [155]. Alterations in circadian rhythms impact various aspects of human cardiac electrophysiology, such as heart rate, QT interval, QT interval dispersion, and ventricular occlusion. Moreover, the effects of aging on circadian rhythm alterations have been documented in heart gene expression. These changes can potentially contribute to cardiovascular dysfunction or disease [156]. Natriuretic peptides (NPs) constitute a group of cardioprotective hormones involved in the regulation of cardiac structure and electrophysiology. They have been shown to modulate atrial conduction and arrhythmogenesis by affecting atrial fibrosis. In aged NPR-C^−^^/−^ mice, the left atrium exhibited the highest levels of atrial stromal fibrosis due to the loss of NPR-C. This loss also leads to a reduced lifespan and accelerates the onset of senescence. Additionally, a shortened atrial action potential duration can increase susceptibility to atrial fibrillation as senescence increases [157]. Aging can regulate atrial and pulmonary vein electrical activity through mechanisms such as mechanoelectrical feedback, imbalances in calcium levels, oxidative stress, and metabolic irregularities. Overall, senescence at the cellular level has significant impacts on the electrophysiology of the heart, potentially resulting in impaired autoregulation at pacemaker sites, impaired conduction function, modified action potentials, and abnormalities in ion channels, consequently increasing the likelihood of arrhythmias.

#### The role of cellular senescence in atrial fibrillation

Atrial fibrillation (AF) is widely recognized as a significant arrhythmia among older individuals, with its morbidity and mortality rates escalating with advancing age. AF pathogenesis primarily involves remodeling of the heart’s electrical system and structural alterations, and aging significantly contributes to the development of electrical and structural remodeling in the atria, resulting in heightened vulnerability to AF. As individuals age, the interplay between reduced cardiomyocyte function, oxidative stress, calcium dysregulation, apoptosis, and atrial myocyte fibrosis induces alterations in both the electrical and structural remodeling of the atria, thereby facilitating the onset and persistence of AF [158]. As individuals age, the normal conduction system of the heart(including the sinusoatrial node, atrioventricular node, and His-Purkinje fibers) experiences infiltration by senescent cells and fibrofatty tissue, as well as collagen, amyloid, lipid, and elastic tissue infiltration. This infiltration consequently modifies the intricate interactions among the ion channels involved in depolarization and repolarization [159]. Additionally, atrial conduction that is slow and inhomogeneous, along with lower atrial unipolar voltage, can contribute to an elevated vulnerability to age-related atrial fibrillation [160]. When the myocardium ages, there are failures in intracellular signaling and cellular processes, resulting in the senescence of cardiomyocytes and their apoptosis. Additionally, oxidative stress impacts the function of the sodium and calcium channels, consequently causing increased conduction and a longer period of inactivity. Myocardial heterogeneity increases as a result of the aging process, creating a setting that easily triggers refractory and triggered activities, resulting in the occurrence of arrhythmias [161–166]. The primary electrophysiological characteristics consist of a shortened refractory period in the atria along with reduced adaptation of action potential timing based on frequency [167, 168]. Aging-induced alterations in delayed rectifier potassium current, or diminished plateau potentials, result in an extended duration of action potentials in the atrial myocardium of aged dogs, as well as modifications in atrial action potentials during advanced age. This can cause attenuated conduction of premature beats, consequently prompting the early occurrence of slow conduction premature beats that may additionally contribute to the development of atrial fibrillation [169]. Previous studies have shown a significant age-related prolongation of P-wave duration and dispersion in older dogs, resulting in a reduction of atrial conduction velocities. A decrease in the depolarizing current ICa-L or an increase in the repolarizing current can contribute to a reduction in the action potential (AP) plateau. Nonetheless, a decrease in ICa-L is likely the primary cause of the diminished plateau potential in left atrial (LA) cardiomyocytes of aged dogs [170]. Additionally, the values of APD90 and effective atrial occlusion exhibited lengthening in elderly RA, whereas they showed shortening in elderly LA [171]. Differences in Action Potentials between RA and LA and the shortening of atrial action potential duration (APD) provide an electrophysiological explanation for the presence of foldback and the development of atrial fibrillation [158]. Senescence of endothelial cells or fibroblasts can lead to remodeling of the atria through the activation of proinflammatory and profibrotic signaling pathways, which increases vulnerability to the development of atrial fibrillation [115]. The aging process is often accompanied by cardiomyocyte apoptosis and necrosis. This is accompanied by elevated levels of peripheral inflammation, reactive oxygen species, and the senescence-associated secretory phenotype. As a compensatory response, there is cellular hypertrophy and an accumulation of extracellular matrix in the periphery. Additionally, fibroblast proliferation and macrophages polarization towards the M1 phenotype further contribute to pathological changes in myocardial tissues, ultimately leading to an increased vulnerability to atrial fibrillation [158]. Cardiomyocyte senescence causes a decline in myocardial contractility and the development of abnormal conduction patterns, leading to cardiomyopathy or arrhythmias. It may further increase the risk of ventricular arrhythmias. The heightened levels of ROS in senescent rat ventricular cardiomyocytes hinder their ability to synchronize with electrical pacing, indicating an elevated risk of arrhythmias [172–174]. Endothelial and fibroblast senescence have been observed to be associated with atrial fibrillation. There is a positive correlation between the expression levels of senescence-related markers p53 and p16 and the severity of atrial fibrillation [175, 176]. In conclusion, there remain several unanswered questions regarding the role of cellular senescence in atrial fibrillation. Future studies should thoroughly investigate its underlying mechanisms, aiming to establish a theoretical basis for the treatment of atrial fibrillation.

#### Effects of targeted cellular senescence-associated therapy on arrhythmias

The combination therapy of dasatinib and quercetin (D&Q) has shown potential effects in anti-aging and clearing senescent cells. Julian U G Wagner et al. showed that 2 months of D&Q treatment in aged mice (18–20 months old) reduced vulnerability to arrhythmia due to age-related reversal of ventricular neural innervation [177]. Meanwhile, quercetin, a type of antifibrotic agent, has demonstrated effectiveness in decreasing atrial fibrosis and the subsequent onset of AF in rodent models. Furthermore, Quercetin may alleviate AF by inhibiting fibrosis of atrial tissues through inhibiting TGF-β/Smads pathway via promoting miR-135b expression [178]. Despite receiving increasing attention, radiofrequency ablation is not entirely effective in preventing high recurrence rates for patients after the procedure. Radiofrequency ablation and traditional antiarrhythmic drugs are insufficient in meeting the increasing treatment demand for older patients with enlarged atria. Currently, the primary treatment approach for atrial fibrillation involves the management of ventricular rate, restoration of sinus rhythm, and prevention of blood clot formation. These treatments primarily aim to alleviate the patient’s symptoms, mitigate complications, but do not address the underlying cause of atrial fibrillation. Several recent studies firmly establish the significance of cellular senescence in the pathogenesis of atrial fibrillation. However, limited studies have been conducted regarding the treatment of arrhythmias, such as atrial fibrillation, through mediating or eliminating cellular senescence. Hence, intervening in the senescence process is likely to create new treatment opportunities for atrial fibrillation (Table 1).

### The role of cellular senescence in cardiomyopathy

In different types of cardiomyopathy, cellular senescence is a key pathologic factor driving disease progression. However, its specific mechanism of action exhibits significant heterogeneity due to etiologic differences. Clinically, diabetes induces cardiovascular aging and inflammation, increasing the risk of cardiomyopathy [179, 180]. Senescent cells secrete senescence-associated secretory phenotype and proinflammatory mediators, including macrophages, T cells, and neutrophils, which eliminate senescent cells through immune cell recruitment [22, 181]. Poor macrophage recruitment promotes the accumulation of senescent cells, leading to tissue damage and organ failure [181]. In diabetic mice, attenuating diabetes-induced cell senescence protects against DCM [182]. Almohaimeed GM et al. showed that Diabetes mellitus can induce cardiac senescence by increasing the expression of senescence markers and decreasing the expression of anti-aging proteins. Treatment with metformin can polarize macrophages towards the M2 phenotype, reduce the expression of p16^INK4a^ and senescence-associated β-galactosidase (SA-β-gal), as well as enhance the expression of anti-aging proteins Klotho and growth differentiation factor-15 (GDF-15), thereby preventing diabetic cardiomyopathy (DCM) [183]. Doxorubicin (DOX), a widely used anthracycline anticancer drug, ultimately induces cardiotoxicity and triggers cardiomyopathy by triggering multiple mechanisms such as oxidative stress, mitochondrial dysfunction, cardiomyocyte apoptosis and necrosis, as well as promoting cellular senescence [184]. A study by Zhuang L et al. revealed that exosomes derived from mesenchymal stem cells pretreated with macrophage migration inhibitory factor (MIF) (exosomeMIF) could act as a promising anti-aging therapeutic agent against doxorubicin-induced cardiotoxicity by delivering long non-coding RNA-NEAT1 to inhibit microRNA-221-3p and activate Sirt2, potentially offering cardiac protection during cancer chemotherapy [185]. Aprocitentan inhibits copper death, oxidative stress, and mitochondrial dysfunction through activation of the SIRT7 signalling pathway and exerts biological effects against cardiac and cellular senescence, providing a novel therapeutic strategy for DOX-induced cardiomyopathy [186]. In dilated cardiomyopathy, myocardial fibroblasts become dysfunctional and senescent [187]. Moreover, telomere shortening is a biomarker of premature CM aging in genetic HCM and DCM cardiomyopathies [188]. The progressive and later DCM onset could be linked with the RyR2-mediated increased fibrosis and premature senescence, eventually causing cell death and further cardiac fibrosis in a vicious cycle leading to further hypocontractility as a major feature of DCM [189]. Cellular senescence promotes disease progression by inducing mitochondrial dysfunction, dysregulated autophagy, and senescence-associated secretory phenotype (SASP), releasing pro-inflammatory factors and matrix remodelling proteases that may exacerbate central myofibrillar fatty infiltration, electrophysiological abnormalities, and ventricular remodelling in arrhythmogenic cardiomyopathy (ACM). TMEM43 haploinsufficiency can activate the DNA damage (DDR) and TP53 pathways, resulting in increased SASP expression and arrhythmogenic cardiomyopathy(ACM). Given that TMEM43 is a nuclear envelope protein and that defects in lamin A/C, another nuclear envelope protein, have been previously found to activate this pathway, it is hypothesised that DDR is a common mechanism by which mutations in nuclear envelope proteins lead to the development of cardiomyopathy [190, 191]. Based on the above relevant studies, cellular senescence in different types of cardiomyopathy is summarized in the table (Table 3).

**Table 3:** Senescent cell types, associated markers, and mechanisms of action in cardiomyopathy.

Research model	Cellular senescence types	Targets or pathways	Mechanism of action	Effects	Refs
Diabetic cardiomyopathy	Cardiomyocyte	SA-β-gal, p16^INK4a^, p53,	• OP treatment downregulates CDK1, GTSE1, and CCNB2 transcriptional expressions. • Inhibition of p53 prevents Cardiac Aging by improving glucose metabolism and angiogenesis through increased HIF-1α protein stability. • D + Q treatment efficiently eliminated senescent cells, rescuing CSCs function, which resulted in functional myocardial repair/regeneration.	Alleviate	[184, 187, 197]
Adriamycin cardiomyopathy	Cardiomyocyte cardiac progenitor cells	p53, p16, p21, DDR, SA-β-gal, Telomere shortening, SASP	• PARP-2 inhibits the expression and activity of SIRT1. • C5aRA downregulates levels of p53, p16, p21. • The pro-survival miR-34a upregulates the expression of its target genes Bcl-2 and SIRT1, and downregulates the acetylation level of p53 and the expression level of p16^INK4a^. • Doxorubicin increased the relative proportion of p16^INK4a+^/myosin^+^ cells at the expense of the p16^INK4a−^/myosin^+^ as well as increased SA-β-gal activity in the left ventricle. • Senolytics can effectively reverse the aging phenotype associated with hCardioids by reducing oxidative stress, aging biomarkers, and SASP, thereby restoring organoid viability, function, cardiac progenitor cell numbers, and cardiomyocyte proliferation capacity. • Knocking down p16INK4A in hCPCs can activate anti-apoptotic and antioxidant pathways via the NF-κB signaling pathway to reverse their senescent phenotype and exert antioxidant effects on aged hCPCs.	Alleviate	[12, 198–202]
Dilated cardiomyopathy	Myofibroblasts cardiomyocyte	p53, SA-β-gal, Telomere shortening, Mitochondrial dysfunction	• Deletion of the Lmna gene in fibroblasts may result in LMNA-associated DCM by inducing double-stranded DNA breaks, activating the DDR pathway, and inducing SASP protein expression. • In vitro, sST2 activated TGFβ signaling through the phosphorylation of the SMAD complex to induce MCF activation and inhibit cellular senescence by the Sirt1/p53/p21 signaling pathway. • Active MnSOD detoxification activity, lacking the normal switch between non-acetylated and acetylated forms, dysregulates mitochondrial physiology during development. • Telomere erosion, cellular senescence, and death characterize aged diseased hearts and the development of cardiac failure in humans. • Changes in sFRP-1 expression during cellular senescence may influence the function of cardiac fibroblasts by affecting the Wnt signaling pathway.	Promote	[200, 203–205]
Arrhythmogenic cardiomyopathy	Fibroblasts	SASP, DDR, SAHF	Mutant LEMD2 leads to remarkable changes in the shape of nuclei with condensed heterochromatin formation, reduced proliferation capacity, and cell senescence in fibroblasts, suggesting the involvement of LEMD2 in chromatin remodeling and premature aging.	Promote	[196]

*CDK1* cyclin-dependent kinase 1, *GTSE1* G two S phase expressed protein 1, *CCNB2* cyclin B2, *D* dasatinib, *Q* quercetin, *CSCs* Cardiac progenitor cells, *shCardioids* human induced pluripotent stem cell-derived cardiac cardioid, *DDR* DNA damage, *SAHF* senescence-associated heterochromatin foci.

### Interaction of cellular senescence with other pathways: from mechanistic synergies to disease amplification effects

Studies have shown that cellular senescence can be implicated by the metabolic profile of the organism, such as mitochondrial dysfunction, nicotinamide adenine dinucleotide (NAD^+^), and hyperglycaemia can drive cellular senescence. Furthermore, cellular senescence can in turn modulate phenotypes related to metabolic function. Both tumor cells and regulatory T cells (Tregs) can influence T cell senescence and their anti-tumor immune function by regulating lipid metabolism dysregulation in conventional T cells. Mechanistically, the MAPK or STAT1/3 signaling pathways synergistically promote the expression of cPLA2α (cytosolic phospholipase A2α) in responsive T cells, leading to alterations in lipid metabolism, accumulation of lipid droplets (LDs), and the development of a senescent phenotype [192]. Initially, Wu et al. identified the key protein PHGDH that regulates serine biosynthesis. To confirm the impact of PHGDH on cellular senescence, the authors employed molecular docking and other experiments, uncovering a series of downstream regulatory mechanisms. Specifically, PHGDH interacts with PKM2. On one hand, it inhibits the PCAF-catalyzed PKM2 K305 acetylation and autophagic degradation; on the other hand, it promotes the p300-catalyzed PKM2 K433 acetylation, thereby stimulating the histone H3T11 phosphorylation activity. These mechanisms collectively regulate the transcription of genes associated with cellular senescence [193]. A prominent hallmark of aging is chronic inflammation. Senescent cells secrete pro-inflammatory factors, namely SASP, which promotes chronic inflammation and can also induce the senescence of normal cells. Studies by Miller et al. have indicated that IL-11 is a pro-inflammatory cytokine. Under normal circumstances, IL-11 is involved in regulating immune responses. However, when its level is excessively high, it can trigger unnecessary inflammation. As age increases, the level of IL-11 in the body gradually rises, and this increase seems to be closely associated with aging-related health issues [194]. Inflammation is typically triggered by the involvement of pattern recognition receptors (PRRs) of the innate immune system. The cGAS–STING pathway, which mediates immune sensing of DNA, serves as a key driver of chronic inflammation and functional decline during the aging process [195]. Recently, the team led by David A. Sinclair from Harvard Medical School, by employing the Inducible changes to the epigenome (ICE) system, discovered that epigenetic information dysregulation leads to aging in mice, and restoring the integrity of the epigenome can reverse signs of aging. This further clarifies that DNA changes are not the sole, nor even the primary, cause of aging; instead, epigenetic changes are the main driving factor for mammalian aging [196]. Epigenetic modifications such as DNA methylation and histone modifications play an important role in the aging process. For example, the study by Li et al revealed the epigenetic metabolic axis that promotes aging by detecting the methylation level and expression level of Elvol2 and applying an artificial intelligence approach to predict the protein structure of Elvol2 and its interactions with substrates [197]. Recently, Christian M. Nefzger’s group at The University of Queensland conducted a comprehensive analysis of chromatin remodelling and transcriptional changes in 22 mouse cell types, revealing a key link between early chromatin accessibility and the ageing process. Histological analyses of both processes identified shared transcription factor binding patterns in genomic regulatory elements, constructed age-related maps of chromatin and transcriptional dynamics, and highlighted the critical role of differences in the abundance of binding sites for the pioneer factor AP-1 and cellular identity transcription factors [198]. In summary, the interactions between cellular senescence and other pathways are complex and far-reaching, and in-depth investigation of these relationships provides new perspectives for understanding the mechanisms of senescence and developing relevant intervention strategies.

## Targeting cellular senescence in cardiovascular diseases: from intervention strategies to heterogeneity-guided precision therapeutics

### Conventional and Emerging Intervention Strategies for Senescent Cells

Although it has been believed for many years that cellular senescence is related to age [12, 199], it was not until recently that reports confirmed that eliminating senescent cells can indeed delay natural aging and premature aging, extend lifespan, and restore organ function [200–202]. An increasing amount of research attests to the accumulation of senescent cells during the progression of a wide array of age-related diseases [203]. The accumulation of senescent cells hastens tissue aging by hindering tissue regeneration and propagating the senescent phenotype through SASP signaling. Consequently, the removal of senescent cells is considered a potential anti-aging strategy [204]. The observation of the positive effects of eliminating senescent cells, along with the apparent absence of long-term negative consequences, has led many researchers to focus on exploring new drugs and strategies to counteract the effects of senescent cells in humans [16]. There is now relevant evidence indicating that the elimination of senescent cells, There is now relevant evidence indicating that the elimination of senescent cells, either through genetic or pharmacological means, has therapeutic effects in several mouse disease models [205]. Strategies involving the targeting of senescent cell removal (Senolytics) through advanced technologies are emerging as innovative anti-aging approaches, with frequently observed drug combinations including dasatinib and the natural flavonoid quercetin [200, 203]. For instance, relevant studies have demonstrated that the synergistic combination of quercetin and dasatinib effectively reduces p16 protein levels, clears ionizing radiation-induced senescent cells, and subsequently improves left ventricular systolic performance in aged mice [206]. Sirtuins are highly conserved enzymes that play a crucial role in the process of aging. They function as deacetylases and ribosyltransferases dependent on NAD^+^. In mammals, there are seven distinct members of sirtuins that are localized differently within cells. These various sirtuins are involved in regulating several essential cellular functions, such as repairing DNA damage, governing the cell cycle, responding to metabolic changes caused by nutrient availability, and preventing neurodegeneration. Moreover, research suggests that sirtuins are associated with longevity and age-related diseases [207–209]. Relevant studies demonstrate the significant role of SIRT2 in the aging process of the primate heart. It has been identified as one of the key proteins that are down-regulated during this process. Additionally, in vitro experiments have confirmed that a deficiency of SIRT2 results in the accelerated senescence of human cardiomyocytes. These findings strongly suggest that SIRT2 serves as a crucial cardioprotective factor for the primate heart during the aging process [1]. SIRT6 exerts various anti-aging effects, encompassing telomere protection, DNA repair, enhancement of genome stability, mitigation of oxidative stress and inflammation, inhibition of endothelial cell senescence, prevention of atherosclerosis, and improvement of glucose metabolism [208, 210]. Epidemiological and experimental studies have established the role of homocysteine in the pathogenesis of cardiovascular disease. It mediates the development of this disease through multiplemechanisms, including the stimulation of vascular smooth muscle cell proliferation, endothelial dysfunction, oxidative damage, enhanced collagen synthesis, arterial stiffness, and increased cholesterol synthesis [211]. Homocysteine also plays a role in promoting atherosclerosis and exacerbating cardiovascular diseases [212]. Folic acid is recognized as a vital vitamin for individuals of all age groups, as it lowers plasma homocysteine levels, acts as an inhibitor of oxidative stress, and preserves DNA stability. Consequently, it has beneficial impacts on cardiovascular health [213]. Folic acid supplementation mitigates cardiac senescence and cellular senescence during aging through the down-regulation of p53-p21 and p16INK4A expression. This attenuation is evidenced by the reduction of left ventricular hypertrophy, prevention of maladaptive left ventricular remodeling, improvement of left ventricular dysfunction, attenuation of interstitial and perivascular fibrosis, and decrease in apoptosis in cardiomyocytes in aged mice [139]. Pharmacologically removing or knocking out senescent cells in mice mitigates cardiac hypertrophy and fibrosis induced by cardiac senescence while also facilitating cardiomyocyte regeneration [145]. Serine biosynthesis originates from glycolysis and plays a pivotal role in averting cardiovascular cellular senescence. Consequently, augmenting serine biosynthesis may serve as a therapeutic approach to foster robust aging [193]. An experimental study substantiates the potential of bromodomain-containing protein 4 (BRD4) as a candidate for treating multiple aging-related diseases [214]. Furthermore, senescence can be regulated by telomerase activation [25]. In addition, the production of the senescence-associated secretory phenotype (SASP) serves as a crucial marker of cellular senescence, influencing the process of senescence through both autocrine and paracrine mechanisms [8]. For instance, TGF-β or IL-1βis crucial constituents of SASP, establishing a self-reinforcing feedback loop in the vascular aging inflammatory response [13]. Medications that target this category of components can hinder SASP and postpone the aging process. Importantly, leveraging the apoptosis resistance intrinsic to senescent cells, scientists developed Bcl-2/Bcl-xL inhibitors (such as ABT-263/ABT-737) that selectively induce senescent cell clearance via targeted pro-apoptotic signaling. This strategy not only remodels the tissue microenvironment but also reactivates dormant stem cells, offering a promising avenue for anti-aging therapies [202, 215]. Following ABT-263-mediated clearance of senescent chondrocytes in osteoarthritis models, transcriptional downregulation of catabolic factors (IL-6, MMP-13) was observed, concomitant with attenuation of cartilage degradation [216]. However, recent studies demonstrate that engineered T cells targeting specific surface markers of senescent cells enable efficient and low-toxicity clearance. In the preceding sections, we have explored the critical role of targeting cellular senescence in various CVDs. To provide a more comprehensive understanding of its potential clinical applications, relevant clinical trial information is summarized in the Table 4. To summarize, the utilization of anti-aging therapy in cardiovascular disease remains limited, with insufficient understanding of its applicability, safety, and extent in human subjects. Consequently, anti-aging research has a significant journey ahead.

**Table 4:** Clinical trials of targeted senolytic therapies and T-cell immunotherapy for cardiovascular diseases.

Disease	Intervention	Study start (actual/estimated)	Status	Phase	ClinicalTrials.gov identifier
Coronary artery disease	Dietary Supplement: omega 3 or vitamin E	2013-10	Completed20	Phase 4	NCT02011906
Heart Failure	Drug: Digoxin 0.125 MG	2025-09-01	Not yet recruiting 100	Phase 2	NCT06240403
Aging endothelial dysfunction	Dietary Supplement: Fisetin	2023-09-25	Recruiting 70	Phase 1/2	NCT06133634
Cardiovascular Disease	Drug: Abatacept 10 mg/kg	2025-09-01	Not yet recruiting 20	Early Phase 1	NCT04344873
Atherosclerosis	Drug: Vitamin A Drug: placebo	2010-05	Unknown status 45	Phase 4	NCT01414972
Atherosclerosis Myocardial Ischemia	Drug: Acetylcholine Drug: Adenosine	2011-01	Unknown status 50	Not Applicable	NCT01162824
Hypertension	Drug: Abatacept	2014-08	Terminated 1	Phase 2	NCT02232880

The data were analyzed using the ClinicalTrials.gov database. https://clinicaltrials.gov/.

### Heterogeneity of senescent cells: challenges and opportunities for precision therapy

Cellular senescence plays a key role in numerous diseases; however, cellular senescence exhibits significant heterogeneity in the form of distinct cellular subpopulations that exhibit unique responses to the aging process [217]. Identifying and understanding these subpopulations is critical to elucidating the complex mechanisms of senescence and developing targeted interventions to mitigate age-related decline. SCS has revolutionised our ability to study cellular heterogeneity by facilitating the unbiased characterisation of the entire cellular component of a tissue, enabling the detection of previously unidentified or infrequent cellular subtypes [218]. For example, Lu et al. identified 11 CD8 T cell subpopulations, each with distinct senescence trajectories and function [219]. Moreover, current research on cellular senescence faces challenges such as the lack of universal markers for senescent cells and the limitations of traditional identification methods. To address these issues, Jing-Dong J. Han’s research team developed the machine learning-based SenCID (Senescent Cell Identification) algorithm, which enables precise identification of senescent cells and assessment of their senescence levels from human single-cell transcriptomic data. SenCID classifies cells into six distinct senescence IDs (SIDs), with notable differences among SIDs in terms of baseline senescence levels, cellular stemness, gene functions, and responses to senolytic treatments. By integrating trajectory reconstruction algorithms, SenCID reconstructed cellular senescence trajectories across various physiological and pathological states—including normal aging, chronic diseases, and COVID-19 infection—using single-cell data from human tissues. Additionally, SenCID was applied to transcriptomic datasets generated by single-cell gene perturbation techniques, identifying genetic factors that either promote or inhibit cellular senescence. These advancements lay a foundation for further exploration of the mechanisms and interventions targeting cellular senescence [220]. Meanwhile, Vidal et al. utilized scRNA-seq technology to perform single-nucleus RNA sequencing on hearts from young (12-week-old) and aged (18-month-old) mice, identifying 12 fibroblast subpopulations. Among these, subpopulations enriched in aged hearts (such as subclusters 2, 3, and 4) exhibited high expression of genes associated with angiogenesis inhibition, endothelial-to-mesenchymal transition(EndMT), and inflammation (Serpine2, Klf4, Tgfbr2). In vitro experiments confirmed that senescent fibroblasts suppress endothelial cell angiogenic capacity by secreting Serpine2 and induce the expression of inflammatory cytokines (IL-6), revealing a molecular mechanism by which fibroblast-endothelial cell interactions via SASP drive cardiac fibrosis. Additionally, the c-Kit^+^ subpopulation of cardiac progenitor cells (CPCs) loses proliferative capacity during aging, promoting myocardial fibrosis through the CXCL12/CXCR4 axis and TGF-β/Smad3 signaling pathway, while endothelial cell senescence triggers vascular dysfunction via the IL-17A/NF-κB pathway [221]. Weiqi Zhang et al. discovered through single-cell analysis of 74 human cardiac tissue samples that the RNA-binding protein ARID5A activates the NF-κB/TBK1 pathway by stabilizing MAVS mRNA, thereby driving cardiac inflammation and aging. Gene therapy-mediated targeted inhibition of ARID5A reversed aging-related phenotypes and improved cardiac function, revealing the ARID5A-MAVS axis as a critical regulator of cardiac aging [222]. Interestingly, single-cell whole-genome sequencing (scWGS) enables comprehensive analysis of the entire genome at single-cell resolution, overcoming limitations of bulk sequencing in detecting rare somatic mutations. Choudhury et al. employed scWGS to investigate somatic single-nucleotide variants (sSNVs) in cardiomyocytes from 56 human donors aged 0.4 to 82 years. Their study revealed a significant age-related increase in sSNVs within cardiomyocytes, with elderly diploid cells exhibiting a sevenfold higher sSNV burden compared to infant cardiomyocytes. This finding underscores the accumulation of genetic mutations as a critical hallmark of cardiac cellular aging [223]. Plasma proteomics is a promising method for identifying clinical biomarkers associated with aging. The study by Ma et al. systematically elucidated the dynamic evolutionary patterns of the plasma proteome during biological aging, identifying 41, 60, and 67 years as critical biological age inflection points. They identified key aging biomarkers with significant biological implications, including GDF15, CXCL13, DPY30, FURIN, IGFBP4, and SHISA5. These findings deepen our understanding of the molecular mechanisms of aging and provide novel insights for developing systemic aging biomarkers and personalized therapeutic targets for age-related diseases [224]. To summarize, Single-cell technology has overcome key bottlenecks in studying cellular heterogeneity during aging, enabling precise subpopulation classification and elucidation of core molecular pathways. These advances lay a scientific foundation for developing innovative therapies targeting specific senescent cell subpopulations.

## Conclusion and outlook

**Fig. 4:**
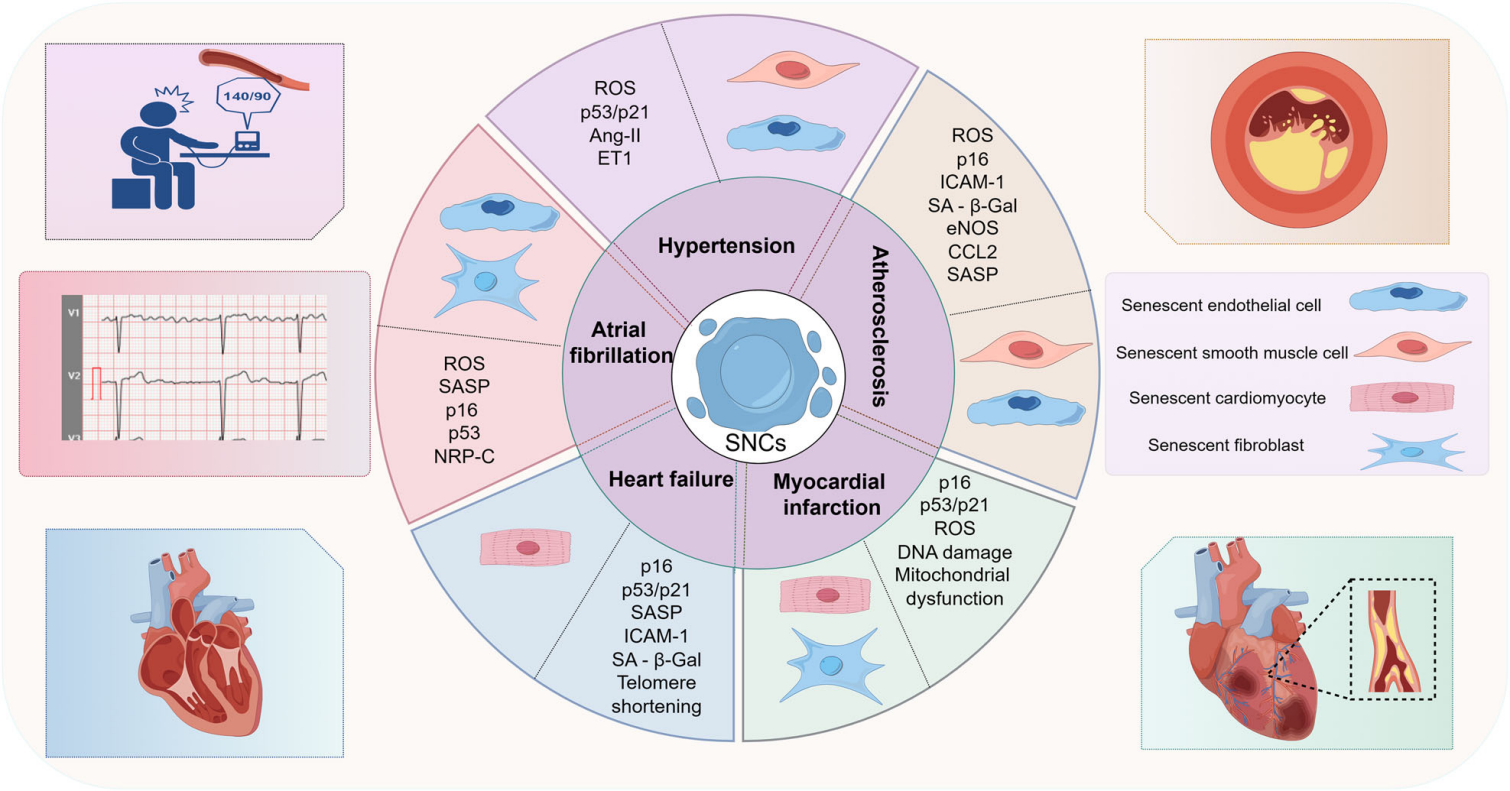
Summary of the association between cellular senescence and cardiovascular disease. This diagram aims to comprehensively illustrate the close connection between cellular senescence and cardiovascular diseases, including hypertension, atherosclerosis, myocardial infarction, heart failure, and atrial fibrillation. It details the specific senescent cell types involved in each disease and highlights the biomarkers closely related to senescence. Furthermore, it visually depicts the pathological features or overall manifestations of these diseases.

The understanding of cellular senescence’s significance in cardiovascular disease is an intricate and crucial research field. A diligent examination of extensive scientific evidence and experimental outcomes has yielded the following principal findings. Firstly, cellular senescence is a multifaceted biological process encompassing various molecular mechanisms and signaling pathways such as mitochondrial dysfunction, heightened inflammatory responses, oxidative stress, disruption of the cell cycle, and telomere depletion. These mechanisms associated with aging mutually interact, resulting in diminished cellular function and consequently elevating the susceptibility to cardiovascular disease. Cellular senescence represents a significant response of the organism to stress and injury. In normal circumstances, the immune system effectively eliminates cellular senescence. However, during aging or chronic injury, senescent cells secrete excessive amounts of pro-inflammatory and pro-fibrotic factors, impairing their removal by the immune mechanism. Consequently, cellular senescence plays a pivotal role in disease progression [25]. Secondly, numerous pertinent studies have demonstrated a direct correlation between cellular senescence and the pathogenesis of cardiovascular diseases. The heightened inflammatory response elicited by senescent cells and alterations in the intracellular environment further amplify the risk of developing cardiovascular diseases, particularly in cases of atherosclerosis, hypertension, and heart failure, where the impact of cellular senescence and endothelial dysfunction is particularly pronounced (Fig. 4). However, there remains limited understanding regarding the implications of various cardiovascular diseases, such as metabolic cardiomyopathy, ventricular arrhythmias, and heart valve disease. Therefore, further investigation into the mechanisms of cellular senescence across different types of cardiovascular diseases is imperative to gain a more comprehensive understanding of their interconnections. Moreover, multiple related studies have highlighted numerous interventions capable of decelerating the cellular aging process. It has further been demonstrated that inhibiting cellular senescence or eliminating already senescent cells could prove effective in averting and treating cardiovascular disease. Several well-known small-molecule drugs, including metformin, rapamycin, resveratrol, and spermidine, have exhibited potential in inhibiting cellular senescence. Consequently, exploring the role of cellular senescence in cardiovascular disease offers fresh insights into the pathogenesis of such conditions and establishes a theoretical foundation for the formulation of novel preventive and therapeutic approaches. However, cellular senescence is a complex process, and senescent cells exhibit a certain degree of heterogeneity in both their morphology and function. The senescence phenotype is influenced by various factors, including different induction modes, source tissues, and cell types. Furthermore, research models of cellular senescence progress through three stages, transitioning from in vitro culture to animal models and then to in vivo studies, imposing significant challenges on the feasibility of developing anti-aging drugs. There has been limited discussion regarding the variations among model studies, and the absence of characterization of naturally occurring senescent cells within the study of senescence has hindered the establishment of a clear definition of the senescent phenotype. To conclude, Further exploration is required to thoroughly investigate the safety, efficacy, and scope of application of these interventions, with the aim of facilitating their translation into clinical practice. For instance, gene editing and stem cell research are being utilized to delve deeper into the molecular mechanisms underlying cellular senescence and to generate potential therapeutic targets.

In conclusion, the role of cellular senescence in cardiovascular disease has garnered significant attention from researchers. It is strongly believed that by understanding and regulating cellular senescence, future treatments for cardiovascular disease can achieve increased precision and efficiency. This has the potential to effectively reduce the morbidity and mortality associated with age-related cardiovascular disease, ultimately maximizing the potential for individuals to lead long and healthy lives.
